# Protocol for measuring cilium length using 3D confocal fluorescence microscopy, CiliaQ software, and a quality control pipeline

**DOI:** 10.1016/j.xpro.2025.104128

**Published:** 2025-10-08

**Authors:** Daniel Burgdorf, Seniz Yüksel, Katharina Sieckmann, Jan N. Hansen, Dagmar Wachten, Nathalie Jurisch-Yaksi

**Affiliations:** 1 Institute of Innate Immunity, Medical Faculty, University of Bonn, 53127 Bonn, Germany; 2 Department of Bioengineering, Stanford University, Stanford, CA 94305, USA; 3 Department of Clinical and Molecular Medicine, Norwegian University of Science and Technology, Erling Skjalgsons Gate 1, 7491 Trondheim, Norway; 4 Institute of Human Genetics, University Hospital, Friedrich-Schiller-University Jena, 07747 Jena, Germany

**Keywords:** Cell Biology, Cell culture, Microscopy

## Abstract

Primary cilia are cellular antennas responding to various biological cues. Cilia undergo substantial changes in length, affecting their ability to sense extracellular signals and trigger downstream effector responses. Here, we present a protocol to image cilia in 2-dimensional cell culture using immunofluorescence staining and confocal microscopy. We describe steps to analyze cilium length using the CiliaQ plugin in Fiji/ImageJ and for using CiliaQ Explorer, a Python-based pipeline that plots CiliaQ-derived data and performs a statistical analysis after quality control.

For complete details on the use and execution of this protocol, please refer to Hansen et al.^1^

## Highlights

• Protocol for immunolabeling and 3D confocal imaging of cilia in cell culture
• Procedure to segment and quantify cilia morphology in 3D via the ImageJ plugin CiliaQ
• Python pipeline to analyze CiliaQ data (plotting, statistics, and quality control)
• Applicable to other ciliated systems, including tissue and organoids

## Abstract (editor highlights)

Publisher’s note: Undertaking any experimental protocol requires adherence to local institutional guidelines for laboratory safety and ethics.

## Graphical abstract

**Figure:**
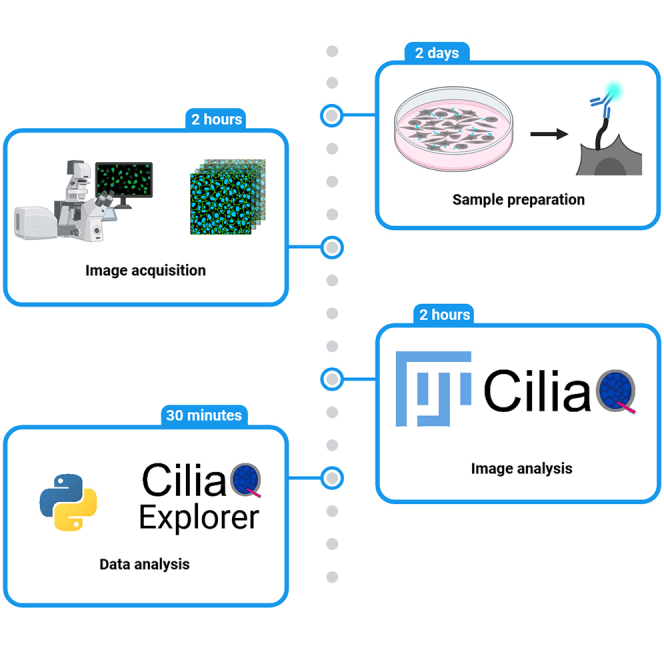

## Before you begin

**Figure 1:**
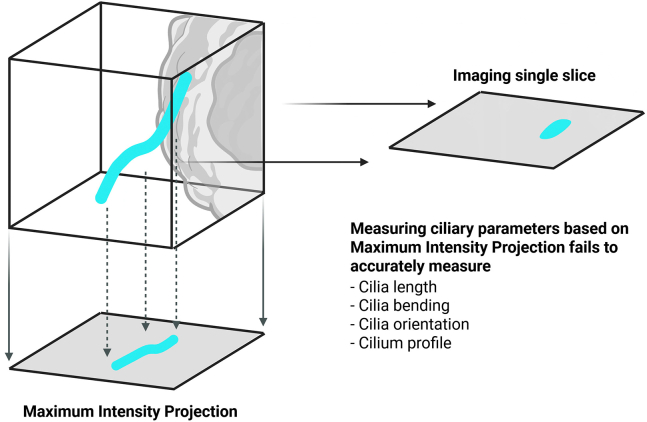
Benefits from performing ciliary analysis on 3-dimensional images Performing ciliary analysis on 2-dimensional images yields a substantial loss of information and leads to inaccurate measurements. When retrieving ciliary information from microscopic images, it is recommended to image a z-stack, consisting of multiple confocal planes. Right: When analyzing only single confocal slice, ciliary signal emerging out of the focal plane is neglected from the analysis, leading to an incomplete mask and thus inaccurate measurements. Below: Compressing a z-stack, to a Maximum Intensity Projection along the z-dimension before analysis, introduces a loss of z-dimensional information during compression, leading to inaccurate measurements of ciliary parameters. This figure was created with BioRender.com.

Cilia are microscopic, microtubule-based organelles that extend from the cell surface and whose membrane is continuous with the plasma membrane. Fluorescence microscopy is the gold standard method to analyze ciliary morphology and behavior.^2^^,^^3^ Since cilia are oriented in space, performing three-dimensional (3D) analysis of fluorescent images is recommended to retrieve accurate measurements (Figure 1). The Fiji/ImageJ^4^ plugin CiliaQ^1^ has been established as a leading tool to segment and measure cilia in 2D-4D fluorescence microscopy images due to its reliable and reproducible outputs. In this protocol, we describe the steps required to label and image cilia of mIMCD-3 cells using a confocal microscope. Of note, due to the high versatility of CiliaQ, the presented procedure can be applied to other microscopy images. This protocol presents results obtained from segmenting cilia based on antibody labeling against Arl13b. Nevertheless, we successfully used CiliaQ to analyze images with different ciliary markers, including acetylated or glutamylated α-tubulin. In addition to ciliary length, all 54 ciliary output parameters from CiliaQ can be analyzed following this protocol, without substantial changes to the workflow. While this protocol describes the analysis of cilia in mIMCD-3 cells, it can be applied to different ciliated cell types, including primary cells, immortalized cell lines, or whole-tissue samples.

### Innovation

This protocol presents an end-to-end and step-by-step workflow for analysis of cilia length, from culturing the cells to data analysis, including immunolabeling, 3D-imaging, and image analysis with CiliaQ. While various cilia immunolabeling methods exist, concise and detailed instructions covering the full process - from sample preparation to quantitative analysis - have been lacking. Here, we provide a robust and reproducible protocol for immunolabeling Arl13b, a lipid-anchored protein in the ciliary membrane. To advance the analytical workflow, we introduce CiliaQ Explorer, a novel Python-based tool that automatically processes CiliaQ output files. Until now, CiliaQ manually extracted meaningful ciliary parameters from the large *.tsv* output files to analyze the data, representing a time-consuming and error-prone workflow. Furthermore, the CiliaQ environment does not enforce or facilitate quality control of ciliary masks, making it time-consuming to explore and easy to overlook improvable cilia segmentation and faulty cilia masks. CiliaQ Explorer replaces manual handling of CiliaQ-derived data with automated statistical analysis and plotting, user-friendly exploratory analysis of ciliary measurements and a simple quality control step. By merging an optimized immunolabeling protocol with automated computational analysis, this workflow offers a reproducible, efficient, and scalable platform for studying cilia morphology, leading to reduced user bias, improved accuracy, and substantially accelerated data processing. This enables streamlined analysis of large numbers of cilia resulting in more robust and comprehensive investigations.

### Cell culture on glass coverslip

**Timing: 2 days**

***Note:*** All steps described in section 1 “cell culture on glass coverslip” must be carried out under sterile conditions using aseptic techniques.

***Note:*** All work should be performed in a laminar flow hood to avoid contamination. Ensure that all instruments and surfaces are disinfected with 70% ethanol or equivalent before use.

***Note:*** Avoid unnecessary exposure of sterile materials to the environment to prevent contamination, and keep in mind that these precautions are essential to maintain cell viability and ensure reproducibility.

***Note:*** All solutions and materials (e.g., coverslips, PBS, Poly-L-lysine, media, pipette tips) must be sterile. Coverslips should be autoclaved or sterilized appropriately before usage.

1. Day 1: Coat the glass coverslips with Poly-L-lysine to improve cell adhesion.
  a. Insert a glass coverslip (e.g., 13 mm) into a multi-well plate.
    ***Note:*** Suitable plates are e.g., a 4-well or 24-well well (both plates have a well diameter of 15.6 mm).
  b. Add 500 μL of 1× Poly-L-lysine (PLL, 0,01% (w/v) in sterile H_2_O) per well, ensuring the coverslip is completely submerged.
  c. Gently push the coverslip to the bottom of the well to ensure proper coating.
  d. Incubate for 30 min at 21°C –23°C.
  e. Remove the PLL solution and wash each well once with 400 μL 1× PBS to remove excess coating reagent.
  f. Remove as much PBS as possible to avoid diluting the cell medium during seeding.
    ***Note:*** The sterile 1× PLL-solution can be prepared prior to the experiment and stored at 4°C (short term-storage) or −20°C (long term-storage, up to 18 months). Avoid repeated freeze/thaw-cycles and excessive exposure to light, as this might reduce coating efficiency.
2. Seed the cells onto the wet coverslips.
  a. Seed the mIMCD-3 cells in sterile DMEM complete medium supplemented with 10% FCS on a PLL-coated coverslip at 100.000 cells in 500 μL per coverslip.
  b. To ensure cell attachment, incubate the plate for 24 h at 37°C and 5% CO_2_.

***Note:*** Drying the PBS-washed coverslips before seeding the cells might reduce the adhesion effectiveness. For maximum binding efficiency, we recommend avoiding drying the coverslips.

***Note:*** Cell count will depend on the cell type and desired confluency. It is recommended to optimize the cell count to achieve high confluency and ciliation rate (mIMCD-3 cells show higher ciliation rates with higher confluency).

3. Day 2: Proceed with serum starvation of the seeded cells.
  a. Replace the medium in each well with 200 μL starvation medium (DMEM complete medium without FCS) to increase the ciliation rate.
  b. Incubate the plate at 37°C and 5% CO_2_ for 12–16 h.

***Note:*** Step 3a is performed to promote cilia formation by omitting serum from the medium. For mIMCD-3 cells, serum deprivation increases the ciliation rate. Depending on the cell type and the study question this step may be omitted or customized.

### Fixation and antibody labeling of cells

**Timing: 3 h**

4. Fix the cells.
  a. Gently wash the coverslips in each well with 200 μl of 1× PBS to remove residual medium or debris.
  b. Fix the cells by adding 300 μL of 4% Formaldehyde (FA) in 1× PBS and incubate them at 21°C–23°C for 20 min.
    ***Note:*** Ensure that the entire well is completely covered to prevent uneven distribution of the fixative.
  c. Wash all fixed wells with 200 μL of 1× PBS three times.
    **CRITICAL:** FA fumes are considered carcinogenic and harmful. Use appropriate protection equipment during handling (wear gloves, use fume hood). All FA waste needs to be collected and discarded following your national or local health and safety guidelines. You may also be required to register your exposure to FA.
    ***Note:*** For each wash, gently add 400 μL 1× PBS along the side of the well to avoid dislodging cells. Ensure PBS completely covers the cells to prevent uneven washing of the media.
    ***Optional:*** At this point, the protocol can be paused for hours up to several weeks, if sterility can be maintained. During the pause, the cells can be kept in 1× PBS and stored at +4°C with a closed and sealed lid to prevent evaporation of the liquid.
5. Proceed with immunostaining.
  ***Note:*** To reduce unspecific binding, a combined blocking and permeabilizing reagent (CT buffer) should be applied prior to and during antibody labeling. Performing both processes simultaneously reduces sample handling and experimental time.
  a. Add 150 μL of CT buffer (0.5% Triton X-100, 5% ChemiBLOCKER in 0.1 M Disodium hydrogen phosphate dihydrate (Na_2_HPO_4_) in 1× PBS, pH 7.0) to each well.
  b. Incubate for 30 min at 21°C–23°C on a horizontally rotating shaker (30 rpm) to provide antibody redistribution.
  c. After incubation, remove CT buffer and directly continue with antibody labeling.
    i. Apply 150 μL of the primary anti-Arl13b antibody dilution in CT buffer (1:1000) to each well.
    ii. Incubate for 1 h at 21°C –23°C on a horizontally rotating shaker (30 rpm), ensuring even coverage of the cells and antibody redistribution across the whole well.
    iii. Wash cells three times with 300 μL 1× PBS per well. Incubate cells in PBS for 1–2 min on a horizontally rotating shaker (30 rpm) before aspirating.
  d. Add 150 μl of the secondary antibody solution (1:500 dilution) diluted in CT to each well.
    i. Incubate for 45 min on a horizontally rotating shaker (30 rpm) at 21°C–23°C in a dark environment to protect the fluorophores from bleaching.
    ii. Wash the cells three times with 300 μl of 1× PBS per well using the same procedure as described above.
      ***Note:*** 4′,6-diamidino-2-phenylindole, dihydrochloride (DAPI, 1:10,000 dilution from (5 mg/mL) stock-solution, Invitrogen) can be used at 500 ng/mL as a DNA counterstain together with the secondary antibody.
      ***Note:*** ChemiBLOCKER can be substituted for other blocking agents available to you (e.g., 4% FBS). If more convenient for you, you may replace CT buffer in 5a-e with an alternative blocking and permeabilization buffer (e.g., 4% FBS + 0.5% Triton-X100 in PBS).
      ***Note:*** CT buffer contains Triton X-100 detergent to permeabilize the sample and improve penetration of antibodies.
      ***Note:*** To dispose CT buffer (containing ChemiBLOCKER and Triton X-100) follow your national or local health and safety guidelines.
      ***Note:*** We recommend using strictly cilia-exclusive, highly specific antigens, such as Arl13b, for ciliary immunolabeling. If additional cellular components are labeled during immunolabeling, generating accurate ciliary masks may be compromised.
      ***Note:*** From the addition of the secondary antibody solution onwards, we recommend covering the plate with aluminum foil to prevent photobleaching due to light exposure.
6. Mount the stained coverslips onto object slides.
  a. Carefully transfer the coverslips with a wide-tip tweezer onto pre-labeled object slides. During transfer, remove excess PBS from the coverslip using a lint-free tissue.
  b. Apply one drop (∼10 μL) of mounting media (Aqua-Poly/Mount or alternative) to the center of each cover slide before placing the coverslip.
  c. Let the slides dry at 21°C–23°C before imaging.
  d. Store slides in a slide storage box at 4°C to maintain fluorescence integrity.

***Note:*** Avoid introducing bubbles under the coverslip by gently placing it onto the mounting medium. Additionally, we recommend using a cut tip that features a wider opening to apply the mounting medium to the slides.

**CRITICAL:** Be sure that the mounting medium is dried and hardened before imaging. If not, ensure adequate airflow during the drying process.

***Note:*** Avoid prolonged light exposure and extreme temperatures during long-term storage. At 4°C, the samples can be stored for several months without significant signal loss. For longer storage, consider re-imaging and assess fluorescence intensity case-by-case.

***Note:*** All required antibodies are listed in the key resources table.

## Key resources table

**Table:** 

REAGENT or RESOURCE	SOURCE	IDENTIFIER
**Antibodies**		

Monoclonal anti-Arl13b antibody (used as 1:1,000 dilution)	Abcam	Cat# ab136648
Goat anti-mouse IgG2a cross-adsorbed secondary antibody, Alexa Fluor 647 (used as 1:500 dilution)	Invitrogen	Cat# A-21241

**Chemicals, peptides, and recombinant proteins**		

Poly-L-lysine	Sigma	Cat# P8920
ChemiBLOCKER	Merck Millipore	Cat# 2170
4',6-diamidino-2-phenylindole (DAPI)	Life Technologies	Cat# D1306
PBS, pH 7.4	Gibco	Cat# 10010023
Formaldehyde, 16% w/v aq. soln., methanol free	Thermo Scientific	Cat# 043368.9M
DMEM/F-12, GlutaMAX supplement	Gibco	Cat# 313310-28
FBS Superior; standardized fetal bovine serum	Biochrom	Cat# S 0615
Sodium azide	Carl Roth	Cat# K305.1
Smoothened agonist (SAG)	Sigma-Aldrich	Cat# SML1314
Triton X-100	Sigma-Aldrich	Cat# X100-5ML
Disodium hydrogen phosphate dihydrate (Na_2_HPO__4__)	Merck Millipore	Cat# 1.06580

**Deposited data**		

Images and CiliaQ output files	Zenodo	Zenodo: https://doi.org/10.5281/zenodo.16891093

**Experimental models: Cell lines**		

mIMCD-3	ATCC	CRL-2123

**Software and algorithms**		

Fiji ImageJ^4^	https://imagej.net/software/fiji/downloads	N/A
CiliaQ Preparator^1^	GitHub: https://github.com/hansenjn/CiliaQ_Preparator	v.1.0.2
CiliaQ Editor^1^	GitHub: https://github.com/hansenjn/CiliaQ_Editor	v.0.0.3
**CiliaQ****^1^**	GitHub: https://github.com/hansenjn/CiliaQ	v.0.1.7
Visual Studio Code	https://code.visualstudio.com/download	N/A
CiliaQ Explorer Script	GitHub: https://github.com/burgdorfd/CiliaQ-Explorer	v.0.1.0
LAS X image acquisition software	Leica Microsystems	v.4.5.0

**Other**		

Glass coverslip (diameter: 13 mm, thickness: 0.3–0.6 mm)	Thermo Scientific	Cat# MEZ 021301.13
Nunclon Delta Surface 4-well plates	Thermo Scientific	Cat# 176740
Nunclon Delta Surface 24-well plates	Thermo Scientific	Cat# 142475
Microscope slides, cut frosted	Epredia	Cat# ISO 8037/1
Aqua-Poly/Mount	Polysciences	Cat# 18606-20
Type F immersion liquid	Leica	Cat# 11513859
Leica STELLARIS 8 confocal microscope	Leica	N/A
HC PL APO CS2 63×/1.40 Oil	Leica Microsystems	N/A
Kimtech Kimwipes Ex-l wipes	Kimberly-Clark	Cat# SKU 33670-04
Tweezer tip 6 mm wide, total length: 105 mm	Schmed	Cat# 62681701

## Materials and equipment

**Table:** Fixative Formaldehyde (FA), 4% w/v

Reagent	Final concentration	Amount
Formaldehyde, 16% w/v	4%	1 mL
1× PBS	75%	3 mL
Total	**N/A**	**4 mL**

***Note:*** Prepare 4% FA freshly before usage on the same day.

**CRITICAL:** FA is considered carcinogenic and harmful. Use appropriate protection equipment during handling (wear gloves, use fume hood). All FA-containing waste needs to be collected and discarded according to your national or local health and safety guidelines. You may also be required to register your exposure to FA.

### Blocking buffer and antibody buffer CT

CT blocking buffer is prepared with 0.5% Triton X-100 (Sigma-Aldrich), 5% ChemiBLOCKER (Merck Millipore) with 0.1 M Disodium hydrogen phosphate dihydrate (Na_2_HPO_4_) in 1× PBS, pH 7.0. The total volume can be adjusted to the required amount.

***Note:*** The prepared CT solution can be stored in a freezer (−20°C) for several months.

***Note:*** ChemiBLOCKER and Triton X-100 are both aquatically toxic and harmful*.* All waste needs to be collected and discarded according to your national or local health and safety guidelines.

**Table:** mIMCD-3 complete medium

Reagent	Final concentration	Amount
DMEM/F-12, GlutaMAX	90% v/v	450 mL
FBS	10% v/v	50 mL
Total	**N/A**	**500 mL**

***Note:*** Media can be stored in a fridge (4°C) for several months if maintaining sterility.

## Step-by-step method details

Negative controls to be performed:

 Unstained cells (skip steps 5c-d.i).

 Primary antibody mixture only (skip step 5c).

 Secondary antibody mixture only (skip step 5d.i).

### Confocal imaging of primary cilia

**Timing: 3 h**

In this step, images of primary cilia are acquired by detecting the signal from Arl13b immunostaining. Multiple z-stacks (3D images) are acquired using a confocal microscope.

***Note:*** We recommend to carefully clean the surface of the cover slip with a lint-free tissue that is dampened with 80% Ethanol (v/v) in H_2_O. This cleans the coverslip from any residual salt crystals and impurities.

1. Start up the microscope.
  a. Switch on the microscope including the control computer and all associated hardware such as lasers, detectors, and cameras. Follow the routine for your individual microscope.
  b. Allow the system a few minutes to initialize.
  c. Start the microscope control software.
2. Preparing the sample and microscope stage.
  a. Choose the objective (e.g. 63× oil immersion) and ensure it is clean.
    ***Note:*** If applicable, before loading the object slides, apply a drop of the recommended immersion liquid (e.g. immersion oil) directly onto the lens.
  b. Carefully place the object slide into the stage.
    ***Note:*** Secure the object slide, in case your stage provides a system for holding the slide tight.
  c. Use brightfield or fluorescence mode to bring the sample into focus using the oculars. Once the correct focus is found, close the shutter immediately to avoid unnecessary bleaching.
    ***Note:*** If you do not use an inverted but an upright microscope, place the immersion liquid onto the cover slip.
    ***Note:*** Depending on your microscope setup, you may use other objectives and immersions, such as 40× objectives, or objectives with different immersion like glycerol or water. We recommend the HC PL APO CS2 63×/1.40 Oil (Leica Microsystems). This 63× objective has a numerical aperture (NA) of 1.40, a working distance of ∼0.14 mm, and is optimized for use with Type F immersion oil (n ≈ 1.518).
3. Set up the image parameters.
  a. Set the image format (e.g., 2048 × 2048 or 1024 × 1024) and zoom (if a laser-scanning confocal is used) to reach a pixel size between 0.05 and 0.1 μm / px.
  b. Ensure that the pinhole size (if adjustable) is set to 1 airy unit.
  c. Set the bit depth to 8-bit.

***Note:*** The Nyquist criterion states that the voxel size should be no larger than half the optical resolution in x-, y-, and z-dimension; e.g., Alexa Fluor 647 imaged with a numerical aperture of 1.4, the maximum lateral resolution is around 290 nm, corresponding to a Nyquist sampling interval of about 145 nm per pixel.

***Note:*** The recommended lateral pixel size of 50–100 nm represents 2–3-fold oversampling relative to the Nyquist criterion. Oversampling results in a finer pixel grid for the ciliary mask, offering increased precision during skeleton fitting in CiliaQ, enhancing morphological accuracy. This comes at the cost of increased file sizes and longer imaging times but improves morphological accuracy.

***Note:*** For purely morphological (length) measures, a bit depth of 8-bit can be storage-space-efficient as it results in smaller file sizes while providing sufficient information to identify cilia. For intensity quantification, a higher bit depth (12-bit or 16-bit) is recommended since this provides a higher dynamic range of intensity measurements.

***Note:*** It might be practical to work with a lower image format size (e.g., 256 × 256) for finding a good region or setting up the z stack, to increase display update speed. Before starting the real acquisition (see below), remember to switch back to the needed image format size and validate that laser and detector settings are also applicable for the larger format size (preventing oversaturation).

4. Adjust the lasers and setting up imaging parameters.
  a. Open the laser control panel in the microscope software.
  b. Activate or select the necessary laser lines / settings based on the fluorophores present.
    i. 405 nm for blue fluorophores (e.g., DAPI) (spectral detection window: 409–478 nm).
    ii. 488 nm for green fluorophores (e.g., Alexa Fluor 488) (spectral detection window: 494–560 nm).
    iii. 647 nm for red fluorophores (e.g., Alexa Fluor 647) (spectral detection window: 660–720 nm).
  c. Set up the detection spectral bands or filter cubes according to the fluorophores present. Depending on the fluorophores, consider recording in multiple sequences (e.g., especially DAPI and Alexa Fluor 488) to avoid crosstalk of emitted signals into the wrong channels.
    ***Note:*** Laser power and detector gain settings depend on the specific laser model, laser age and microscope setup, including optics and detector characteristics. We recommend adjusting the laser intensities and detector gains to the individual microscope setup to obtain the best signal-to-noise ratio.
    ***Note:*** For the STELLARIS 8 microscope setup, we recommend the following laser sources: Diode 405 (405 nm), Argon (458 nm), DPSS 561 (561 nm) and HeNe 633 (633 nm). Alternatives, as long they are comparable, may also be applied to follow this protocol.
    ***Note:*** If you need to understand spectral overlap of fluorophores or find good spectral band settings or fitting filter cubes, you may find the https://www.fpbase.org/spectra/ webpage helpful.
    ***Note:*** When comparing multiple samples, make sure that all samples are stained with the same fluorophore and imaged with the same settings. Ciliary measurements will be influenced by the image resolution, which depends on the emission wavelength of the fluorophore and the imaging settings (objective, numerical aperture, z-step interval, and pixel size, the latter being determined by zoom factor and format (e.g., 1024 × 1024 pixel)).
    **CRITICAL:** If you aim to study ciliary fluorescence intensity, it is mandatory that you image all groups of samples to be compared with the same settings for the channels from which you want to quantify intensity.
  d. Adjust laser intensity, starting from low power and increasing gradually while monitoring the fluorescence signal to avoid oversaturation.
  e. Depending on the microscope, eventually also adjust the detector gain, scan speed, and settings for line averaging or line accumulation to optimize the signal to noise ratio.
    ***Note:*** We recommend a line averaging of 2 to improve signal-to-noise ratio and to use a pixel dwell time of circa 3.2 μs/pixel.
    ***Note:*** An approach thriving for optimal image quality and speed: First, try to increase laser power to a moderate range to improve signal while avoiding photobleaching; second, consider lowering the scan speed or activate line averaging or line accumulation; third, consider increasing the gain.
5. Setting up a z-stack.
  a. Enable “Z-Stack” mode to perform multi-plane imaging.
  b. Use the focus knob to find the upper and lower focal planes of your sample, setting these as the start and end points for the z-stack.
  c. Set the step size (z-step interval) based on the desired resolution. Smaller steps provide better axial resolution but increase acquisition time and data size.

***Note:*** For cilia length quantification, we recommend a pixel size of 0.05–0.1 μm and z-step interval of 0.5–0.80 μm. These settings focus on a good tradeoff between acquisition speed and sufficient accuracy.

6. Capturing the z-stack image.
  a. Perform a live preview of the imaging area to verify that all settings, including exposure and focus, are correct.
  b. Click “Start” or “Acquire” to initiate automatic imaging through the defined z-stack range.
  c. Once the imaging is complete, save the data set in an appropriate format (e.g., .lif or .tiff) for further analysis. We recommend saving the data in the original format of the microscope to retain all metadata.

***Note:*** If you notice photobleaching, reduce laser power and / or shorten exposure times while keeping a balance between signal intensity and sample longevity.

7. Shutting down the microscope.
  a. Ensure all acquired data is saved and backed up before shutting down the system.
  b. Shut down the software and microscope according to your individual microscope-specific routine.
  c. Clean the objective lenses and microscope stage. If e.g. immersion oil was used, wipe it off using lens paper and an appropriate cleaning solution.
  d. Properly store or dispose of samples according to lab protocols.
8. Save all metadata associated with the experimental setup during image acquisition.

***Note:*** To streamline saving experimental metadata into one document, we provide an excel sheet (Table S1) with guidelines on which information should be recovered for downstream analytical processes.

### CiliaQ analysis

**Timing: 2 h**

In this step, we describe a 4-step workflow to segment and analyze cilia, beginning by ciliary segmentation using the CiliaQ Preparator, followed by editing the ciliary masks (CiliaQ Editor), measuring the masks (CiliaQ) and plotting the measurements (CiliaQ Explorer).

***Note:*** If the used device already has Fiji ImageJ installed, please proceed with step 9b.

9. Download the Fiji (Fiji is just ImageJ) software version suitable to your operating system from https://imagej.net/software/fiji/downloads.
  a. Open the executable Fiji ImageJ file from the installation directory and navigate to “Help” *>* “Update”.
  b. Via “Manage Update Sites”, select “3D Image Suite” and “CiliaQ” and press “Apply and Close”.
  c. Press “Apply changes” to start installation of CiliaQ, then restart FIJI ImageJ.

***Note:*** The first step of measuring ciliary parameters via CiliaQ is segmenting the cilia signal. Segmentation is the process of defining foreground (i.e., cilia) and background signal in the ciliary marker channel by applying an intensity threshold. This threshold value can be selected either manually or by one of various available histogram-based algorithms. To ensure accurate measurement, it is important to select a thresholding algorithm that delineates the cilium as its true boundary, without assigning ciliary signal to the background or neglecting genuine ciliary signal.

**Figure 2:**
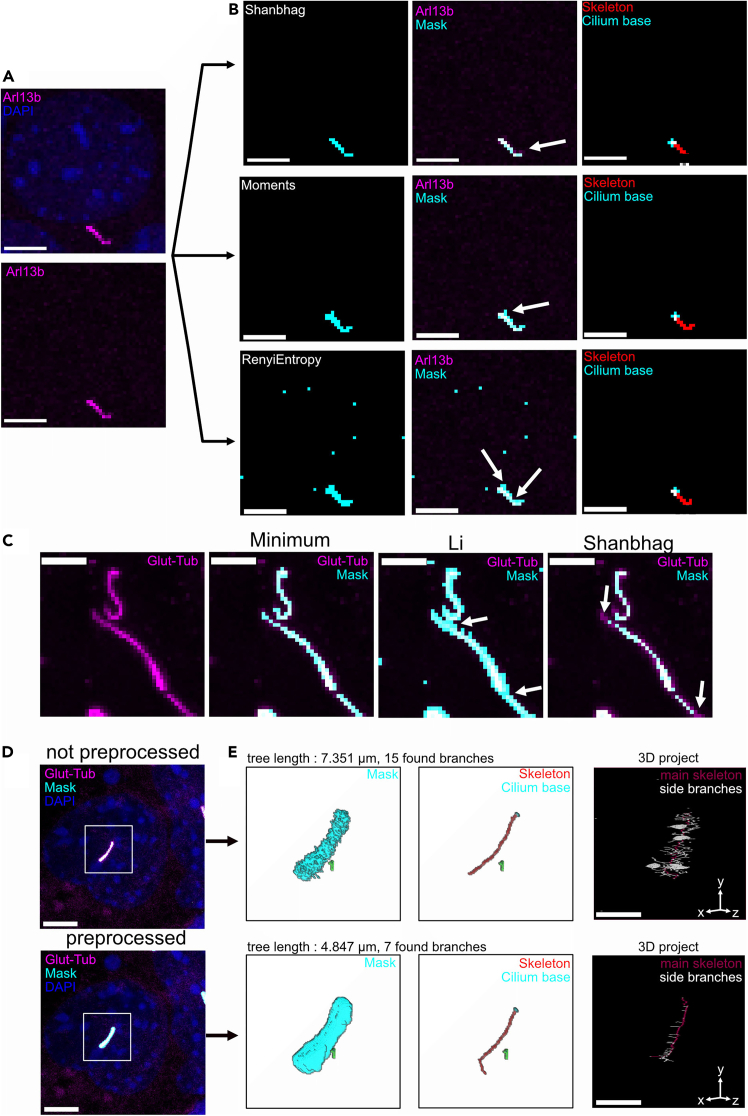
Segmenting ciliary signal including common pitfalls of over- and undersegmentation (A) Maximum projection of 3T3 cells stained with DAPI and anti-Arl13b. Scale bar: 5 μm. (B) Common pitfalls during ciliary segmentation. Shown are ciliary masks (left) and overlay of masks and Ar13b signal of an inset (right). The Shanbhag threshold algorithm yields a threshold that does not detect all pixels of the cilium (“undersegmented result”), yielding to a small gap in the ciliary mask, which would result in the cilium being split into two separate objects in the CiliaQ results. Both, Moments and Renyi Entropy algorithms, produce oversegmented results, resulting in masks that expand beyond the Arl13b signal and produce branches attached to the mask. If these branches are smaller than the overall ciliary shape, they may however not influence length measurements as these are done by following the major “branches” (see right, from CiliaQ output SKL.tif file). (C) Common pitfalls for cilia segmentation demonstrated on zebrafish head, stained against polyglutamylated tubulin [GT335] (top left). Ciliary masking based on an intensity threshold calculated with the Minimum algorithm produces a thin mask, where the continuous cilia are detected, accounting for complete cilia (top right). Using the Li algorithm produces masks that expand beyond the ciliary signal, accidentally fusing close cilia (bottom left). Using the Shanbhag algorithm results in discontinuous masks which do not account for the complete glutamylated Tubulin signal and would result in detecting all cilia as multiple objects or not detect the whole ciliary length. Scale bar: 5 μm. (D) Segmentation of ciliary signal with and without image preprocessing. A high-resolution image (voxel depth = 0.25 μm/px, pixel dimensions = 0.046 μm/px for both pixel height/width) from Arl13b-GFP expressing 3T3-L1 cilia-iAPEX cells was segmented using the histogram-based Renyi Entropy algorithm. For visualization purposes, all images are displayed as maximum intensity projection, although the analysis was carried out in 3D. Top: Segmentation of ciliary signal without any prior preprocessing steps. Bottom: Segmentation of ciliary signal in an image that has been preprocessed using a Gaussian Blur filter (σ = 2.0) and Subtract Background function (50 px) before employing the Renyi Entropy algorithm. Scale bar: 5 μm. (E) Effect of image preprocessing on ciliary masks. Without preprocessing, CiliaQ produces a branched ciliary mask with uneven outlines. This is shown by a 3D render of the ciliary mask (top left) and a 3D Project of the skeletonized mask (top right) using the 3D Project function built into Fiji ImageJ (z-step interval = 0.25 μm, scale bar: 2 μm, upscaling of the ciliary mask layer by factor 3 in x-, y- and z-dimension). Ciliary branches are substantially reduced by image preprocessing using a Gaussian Blur and Subtract Background, shown by a 3D render of the ciliary mask (bottom left) and a 3D project of the ciliary skeleton (bottom right). Without preprocessing: tree length = 7.351 μm, cilia length = 5.035 μm and 15 branches; with preprocessing: tree length = 4.847 μm, cilia length = 4.139 μm and 7 branches. Additionally, Image preprocessing enables detection of a bend cilium, shown by a 3D render of the ciliary skeleton without (top middle) versus with image preprocessing (bottom middle). While the noisy pixels will influence intensity measurements, ciliary length measurements may still be mostly accurate with both masks (bottom panel), albeit less accurate for the left compared to the right mask.

***Note:*** Examples of acceptable and improvable cilium masks can be found in Figure 2 and in the CiliaQ wiki. The acquired images are analyzed using the newest version of CiliaQ (v0.1.7) in ImageJ.

10. Select a suitable threshold algorithm for segmentation in a maximum projection (In this step we mimic CiliaQ processing manually, which allows us to see live how certain parameters that we can set in CiliaQ affect the image preprocessing and segmentation).
  a. Open the raw microscopy file in Fiji acquired in step 3–8 and save it as .tif-file via “File” *>* “Save As” *>* “Tiff”.
  b. Open the saved .tif-file and create a Maximum Projection via “Image” *>* “Stacks” *>* “Z-Project”*.* Include all slices and select “Max Intensity” as the “Projection Type”.
  c. Split the channels of the Maximum Intensity Projection via “Image” *>* “Color” *>* “Split Channels”
  d. Duplicate the ciliary marker channel (i.e., depicting Arl13b staining) via “Image” > “Duplicate”. Keep the duplicated image as a backup for merging both images later in step 10i. Continue with the Arl13b channel image.
  e. If your image contains a lot of defocus or background labeling signals, you can consider testing whether the “Process” > “Subtract Background” option reduces them. Use the preview option to test different radius values. If you decide to apply the method as you find it supportive, note down the radius and click “Apply”.
  f. If ciliary signals are fuzzy / noisy, you can consider using a “Process” > “Filters” > “Gaussian Blur” to smoothen the cilia. Use the preview options and test different radius values. If you decide to apply this option, note down the radius and click “Apply”.
  g. Test thresholding algorithms via “Image” > “Adjust” > “Threshold” on one of the duplicated ciliary marker images (see step 10d) by selecting a thresholding algorithm and observing what is previewed as detected pixels in the channel image. If you find a threshold suitable to produce ciliary masks, click “Apply”.
  h. Ensure that the image is 8-bit depth via Image > Type > 8-bit.
  i. Overlay the ciliary mask with the duplicated marker image using “Image” > “Color” > “Merge Channels”.
  j. Display the resulting 2-channel image as a Composite using the Channels-tool (“Image” > “Color” > “Channels Tool”). You should be able to visually inspect both the segmented and unsegmented channel simultaneously to finally confirm segmentation quality. If the segmentation quality is not good enough, go back to step 10d and try to repeat the steps adjusting the values and algorithm you chose to improve.

**CRITICAL:** Within a single dataset, make sure to use a consistent threshold algorithm across all compared images to ensure consistent segmentation.

**CRITICAL:** Make sure to check segmentation quality via maximum projections (step 10) for as many images as feasible within your dataset.

***Note:*** Depending on the ciliary signal characteristics, the determined threshold value may vary across the thresholding algorithms, potentially creating masks that do not fit the ciliary signal.

***Note:*** Threshold values that are too low include substantial background signals into the ciliary masks, leading to false-positive objects (over-segmentation). Moreover, low threshold values can lead to accidental fusion of ciliary masks in close vicinity (Figure 2).

***Note:*** Excessively high thresholding might exclude dimmer segments of the cilia from the mask and, thereby, lead to incomplete mask coverage, also producing false results (under-segmentation).

***Note:*** Should your data set contain more than six images, you may perform this testing step only for selected images representing the most extreme image quality in your data set (e.g., images with highest and lowest signal to noise ratio). It is important that you ensure that settings are applicable to most images.

***Note:*** You will also investigate segmentation quality in later steps. You might need to go back to step 10 if you realize only later that your selected settings were not suitable for many images.

***Note:*** To help identify the best algorithm for segmentation, we recommend duplicating the ciliary marker and remerging the images after thresholding (step 10d, 10h-i). However, this is not crucially required by the CiliaQ workflow.

***Note:*** If no implemented threshold fulfills the quality requirements, try selecting a custom threshold value or consider a hysteresis threshold algorithm at step 10. The hysteresis thresholding that can later be applied in CiliaQ Preparator segments the image based on two threshold algorithms or values. The concept behind this method is to select one “low”, oversensitive threshold algorithm, which detects all cilia-related pixels but also noisy background pixels, and a “high”, low-sensitivity threshold algorithm, which detects at least one pixel in each cilium but no pixels in any background or noise region. Both thresholds are then combined in the hysteresis thresholding – only those pixels are considered cilia pixels if they exceed the low threshold and are connected to pixels exceeding the high threshold. Conceptually, this method could also be imagined as finding cilia with a high threshold and then expanding the masks to cover the whole cilium within the low threshold region.

***Note:*** If still no implemented threshold provides a good segmentation, consider trying the Canny3D edge detection method in CiliaQ. This method cannot easily be tested manually and needs to be tested by running CiliaQ Preparator and inspecting the segmentation output, as explained below.

**Figure 3:**
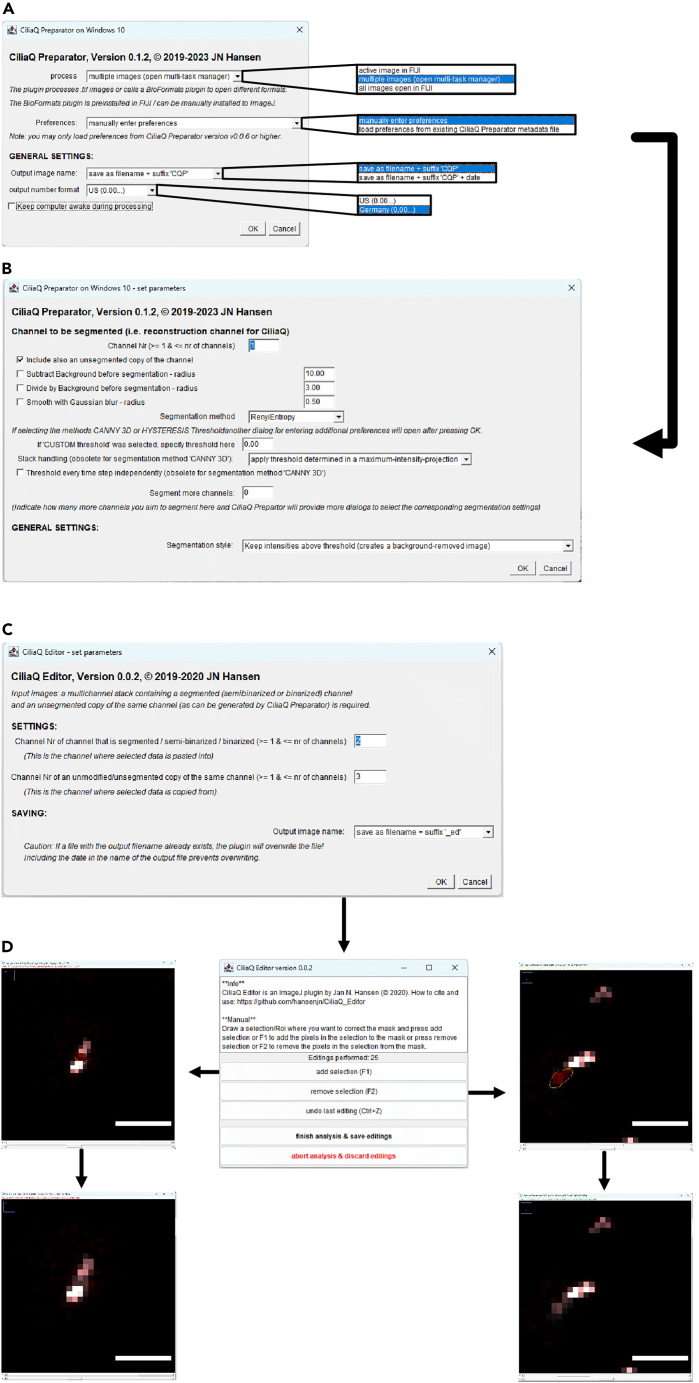
CiliaQ Preparator and Editor workflow (A and B) The CiliaQ Preparator includes two setup windows that allow selecting preferences regarding the CiliaQ Preparator data handling (A) and segmentation (B). (C) CiliaQ Editor setup dialog, that allows selecting preferences. (D) CiliaQ Editor dialog for manual mask editing. Upon selecting a Region of Interest (ROI) using the lasso tool, the dialog allows adding and removing the area to the ciliary mask. A detailed explanation of the CiliaQ workflow also can be found on the CiliaQ Youtube channel.

11. Segment the acquired images using CiliaQ Preparator (Figures 3A and 3B).
  a. Open CiliaQ Preparator in Fiji via “Plugins” > “CiliaQ” > “CiliaQ Preparator” v0.1.2.
  b. Specify how to feed the data into CiliaQ Preparator. If you prefer to load multiple files via a file dialog, select process: “multiple images (open multi-task manager)” *to* manually include multiple images into the analysis.
  c. Enter the CiliaQ-Preparator settings that suited your image (Figures 3A and 3B). Once finished, click ”OK”.
    i. “Channel Nr (>= 1 & <= nr of channels)”: Defines which channel of the image contains the ciliary marker
    ii. “Include also an unsegmented copy of the channel”: Select to include the original and unsegmented image into the CiliaQ Preparator output file.
    iii. “Subtract Background before Segmentation-radius”: Select to introduce a Subtract Background operation before segmenting the image in case you used it in 10e.
    iv. “Smooth with Gaussian Blur - radius”: Select to preprocess the ciliary marker channel by smoothing the signal by introducing a Gaussian Blur in case you used it in 10f.
    v. “Segmentation method”: Specify the threshold algorithm that was determined in step 10g.
    vi. “Select Segmentation style”: Specifies properties of the mask. Leave at the default “Keep intensities above threshold (creates a background-removed image)”. This will create a masked image where intensities in the marker channel are preserved. If you record with a Leica microscope that has near-noise-free detection and produces images with background pixels with intensity values of 0, use the option “Set intensities above threshold to maximum possible intensity (creates a binary image)”.
  d. In the appearing dialog, select your files to be analyzed by clicking “select files individually”. Once finished, click “start processing”.

***Note:*** CiliaQ Preparator features additional methods to feed data into the software. Alternative to “multiple images (open multi-task manager)”, CiliaQ can be applied to the “active image in Fiji” or to “all images open in Fiji”.

***Note:*** Although CiliaQ can be employed on 2D data like Maximum Projections, we highly recommend using CiliaQ on 3D images like confocal z-stacks. Deriving ciliary measurements from a single focal plane might introduce false results, since the cilia might extend further out of the plane, which would not be picked up (See Figure 1).

***Note:*** CiliaQ can load the segmentation preferences from an existing CQ.txt file. To reproduce a past CiliaQ Preparator-Setup, select Preferences: “load preferences from existing CiliaQ Preparator metadata file”. Make sure that the metadata file was produced by the CiliaQ Preparator v0.0.6 or higher.

***Note:*** If image preprocessing via the “Subtract Background”, “Divide by Background” or “Gaussian Blur” operation is necessary should be determined beforehand on the created maximum projection.

***Note:*** Small artifacts created due to slight over-segmentation are acceptable, as long as they are filtered out during minimum cilium size selection (step 11).

12. Refine the ciliary mask using the CiliaQ Editor (optional) (Figures 3C and 3D).
  a. Open the “…CQP.tif” file that is generated in step 11 and contains the ciliary masks in Fiji ImageJ.
  b. Open the CiliaQ Editor software in Fiji via “Plugins” > “CiliaQ” > “CiliaQ Editor”.
  c. Follow the instructions and specify the channel number of the segmented and unsegmented channel (Figure 3C).
  d. Use the freehand selection tool in Fiji to draw a ROI that should be corrected. If the ROI should be added to the mask layer, press “add selection” (F1) or to remove the ROI from the mask layer, press “remove selection” (F2). Once finished, press “finish analysis and save editings”. (Figure 3D).

***Note:*** Depending on the chosen settings and segmentation parameters, the generated masks could vary in quality (Figure 2). Before you continue, visually inspect the mask that was generated in Step 11–12. Visual inspection of the segmentation quality is crucial to further ensure high-precision measurements.

**CRITICAL:** Before proceeding, ensure high quality segmentation by visually inspecting if the ciliary masks fit the signal of the cilia with a high quality. The precision of all measurements depends on the segmentation quality.

***Note:*** We recommend choosing a segmentation method that minimizes artifacts. While CiliaQ Editor enables precise adjustments of ciliary masks, removing artifacts using CiliaQ Editor can be time-consuming.

13. Analyze the prepared masks using CiliaQ.
  a. Set the CiliaQ parameters.
    i. Select “multiple images (open multitask manager)” to include multiple images into the analysis via a file dialog.
    ii. Select “output number format” to “US (0.00)”.
      ***Note:*** Similar to step 11, CiliaQ can reproduce the analysis by reading the preferences from a previous CiliaQ output file (CQ.txt). If favored, select “Preferences”: “Load Preferences from existing results file (“…CQ.txt”)”
      ***Note:*** CiliaQ is also capable of quantifying signal from additional channel within the ciliary masks (“Channel A″,”Channel B″). This feature is valuable when additional target proteins are included in immunostaining and imaging, enabling quantifying protein localization to the cilium.
    iii. Under “channels” > “reconstruction”, insert the channel-ID of the ciliary channel in the image that was generated in step 11 and 12 *(…CQP.tif).*
      ***Note:*** If the data does not contain signal in channel A or B, we recommend keeping the default setting for Channel A/B in step 13a.iii.
    iv. Set the minimum cilium size depending on your resolution. For lower resolutions of 0.1 μm/px, use 10 voxels (default) and for higher resolutions of e.g., 0.0527 μm/px, use 70 voxels.
      ***Note:*** Consider that pixel size is a length and cilium size an area, which is why if you decrease the pixel size you should increase the minimum cilium size exponentially (1/2 of the pixel size = use a minimum cilium size multiplied by at least 2ˆ2 = 4),
      ***Note:*** The minimum cilium size depends on the magnification used during image acquisition. The indicated size determines the minimum size of an object in your segmented channel that you consider a cilium – if you have non-ciliary objects in the segmented channel that are smaller than the cilia you can gauge their size in pixel and adjust this threshold, so they are removed. For lower magnifications, make sure to use a lower minimum cilium size.
    v. Select “Increase range for connecting cilia” and “Determine skeleton-based results (e.g., length)” and additionally exclude “cilia touching x or y or z borders”.
    vi. Introduce a gentle Gaussian Blur on XY-dimensions of the image before skeletonization. Set “gauss filter XY” and sigma to, e.g., 0.5. This can lead to smoother ciliary skeletons and avoid deformed skeletons due to noisy pixels at the border of ciliary masks.
  b. Once applied, load the output files from CiliaQ-Preparator (ending with _CQP.tif) into the CiliaQ analysis and select “start processing”.
    ***Note:*** If CiliaQ Editor was used to refine ciliary masks, the preprocessed file contains a *_CQPed.tif* ending. Make sure to feed the *_CQP_ed.tif* file instead of the *CQP.tif* file into the CiliaQ Analysis.
    ***Note:*** CiliaQ displays the analysis progress during the analysis. Make sure not to close CiliaQ or Fiji during the analysis.
    ***Note:*** For more detailed technical information about CiliaQ, please refer to the CiliaQ publication^1^ or the CiliaQ GitHub page.

### Data analysis

**Timing: 30 min**

In this section, the protocol describes the analysis pipeline downstream of CiliaQ, including condition pooling, quality control, statistical testing, and plotting, which is achieved by the CiliaQ Explorer pipeline, a python-based script (Jupyter Notebook). Along with this protocol, the CiliaQ Explorer Notebook also includes step-by-step instructions to facilitate a smooth and reproducible analysis workflow.

**Figure 4:**
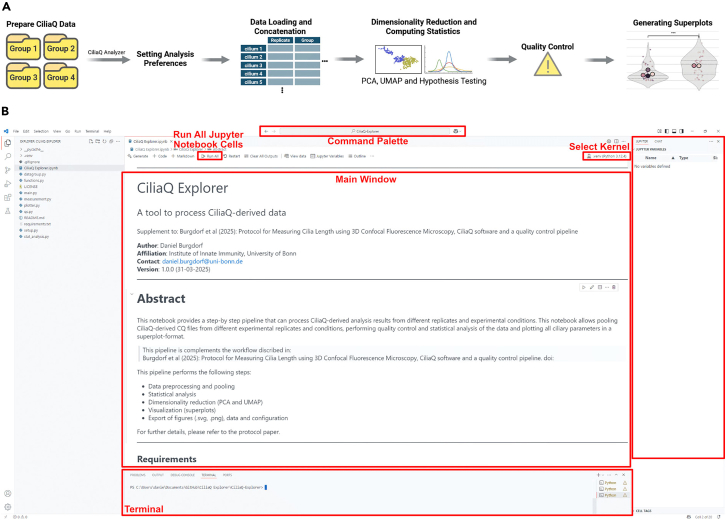
Overview of the CiliaQ Explorer Workflow and the VS Code interface (A) From left to right: The CiliaQ Explorer requires organized CiliaQ-data, with one experimental condition per folder. Following the setup of relevant preferences, the ciliary data from all replicates is concatenated. Based on the setup preferences, the CiliaQ Explorer code computes the statistical analysis, quality control and superplots for the measurements. (B) The Visual Studio Code interface, displaying the CiliaQ Explorer Jupyter Notebook.

**Figure 5:**
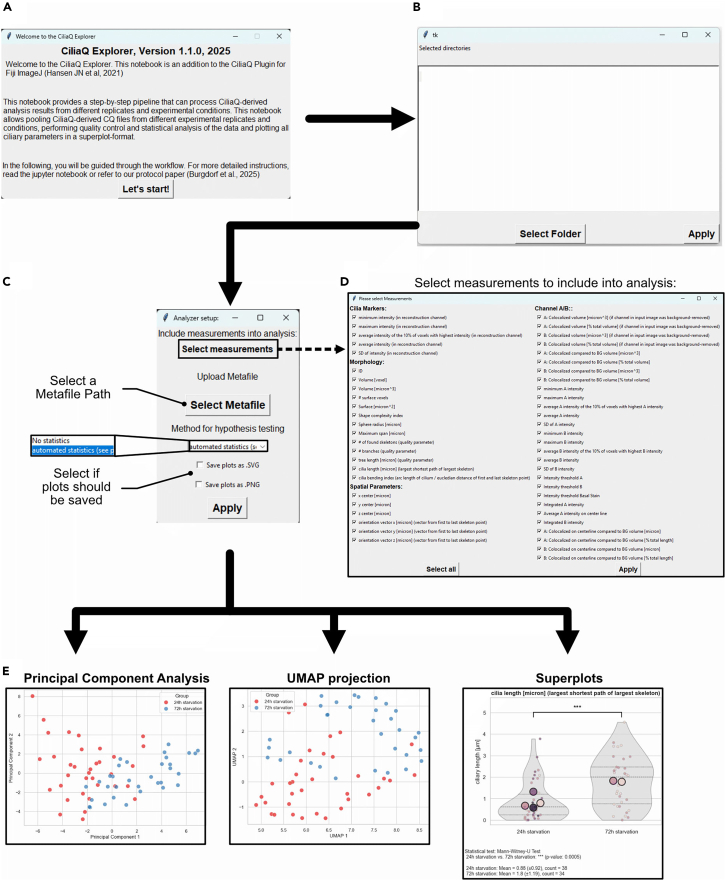
CiliaQ Explorer workflow (A–D) When running the Python script, few dialog windows will open and include a short description of the version (A), a dialog to select experimental folders (B) and the Explorer setup dialog (C). The setup dialog allows selecting all measurements that are included in the analysis (D), uploading a metafile (Table S1), select if statistics should be computed and in which format the data should be saved. (E) Shown are multiple outputs of the CiliaQ Explorer, including a PCA- and UMAP plot and a superplot for each CiliaQ-derived measurement.

14. Plot, perform quality control and run statistics on your data using the python-based CiliaQ Explorer Pipeline. (Figures 4A and 5A–5D).
  a. Install Microsoft Visual Studio Code (VS Code) and create the environment.
    i. Download and install the latest version of VS Code for suitable for operating system from https://code.visualstudio.com/download.
    ii. From GitHub: https://github.com/burgdorfd, download the complete CiliaQ Explorer data folder.
    iii. Download and install the Python software (v3.12.4) from https://www.python.org/downloads/. Make sure to select the option “Add Python to PATH”. Otherwise, Python might not work as intended.
    iv. Install the Python and Jupyter Extension into VS Code via the Extension manager of VS Code.
    v. Open VS Code, open the CiliaQ Explorer Folder via “File” > “Open Folder”.
    vi. Open the Command Palette (Figure 4B) and create a virtual environment (venv) in VS Code by running the “Python: Create Environment” command in the command palette.
    vii. Select “Venv” and choose the downloaded Python software as Interpreter for the virtual environment.
    viii. Install dependencies from the requirements.txt file.
      Once successfully created, choose “.venv” under “Select Kernel” (Figure 4B).
  b. For each experimental condition (“group”), create a dedicated folder on your device that contains all CiliaQ-derived data from each replicate within that condition.
    ***Note:*** For new Python users, the Visual Studio Code webpage for Python provides tutorials for getting started and learning Python.
    ***Note:*** We recommend working in a virtual environment, since it encapsulates the Python working environment. Installing the dependencies in the system environment can lead to version conflicts between packages and compromise reproducibility across different analysis runs. For more information, Microsoft’s explanation of Python environments might be helpful.
    ***Note:*** The folder names determine how experimental conditions are labeled in output plots. Name the folders carefully by using descriptive, short names following a consistent nomenclature across folders.
  c. Run the CiliaQ Explorer in VS Code (“Run all”) (Figure 4B).
    i. In the appearing “Open Directories” dialog, stepwise select the CiliaQ-data containing group folders. Once finished, click “Apply” (Figure 5B).
    ii. In the setup window, select the CiliaQ-derived measurements to be analyzed by clicking “Select measurements”. To select all measurements, select “Select all” (Figure 5D). Optionally, upload a metafile (Table S1) to pass experimental metadata into the pipeline via “Upload metafile”. Finally, select if statistics should be computed and specify the file format for saving the generated plots.
    iii. Read the quality control dialog carefully. To continue with the analysis, select “Confirm”.
    iv. The code generates a series of plots that are described in further details in the expected outcomes section.
    v. Inspect the created plots that are stored as .png and .svg files to the path of the first group. (Figure 5E).
      **CRITICAL:** Read the quality control dialog and the qc.txt file carefully. The CiliaQ Explorer performs a multi-step quality control of the pooled data and detects certain types of outliers throughout the data such as highly branched or excessively long cilia, which indicates that one should validate that outliers are not segmentation errors, accidentally applied wrong settings, or coming from inconsistent image data.
      ***Note:*** CiliaQ Explorer QC is limited and cannot replace a manual inspection of the accurateness of segmentation, detected cilia, or wrong settings (like minimum cilium size threshold too high). To ensure high quality segmentation, we recommend revalidating the masks of the identified cilia visually as well.
      ***Note:*** Pooling and comparing CiliaQ data require all analysis to be performed on the same CiliaQ version. When the data is pooled via the CiliaQ Explorer, use of the same CiliaQ versions throughout all data is checked by default.

## Expected outcomes

**Figure 6:**
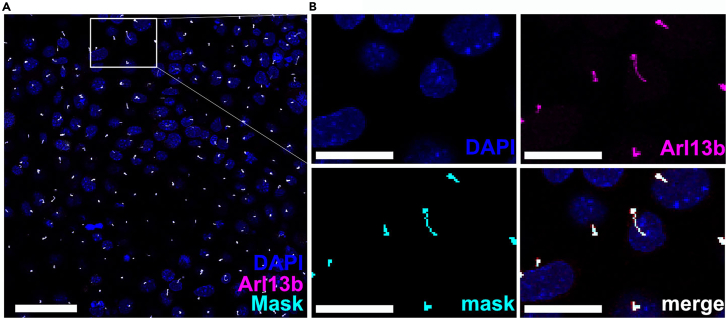
mIMCD3-cells were immunostained with Arl13b to label the cilia and DAPI for the nuclei The image was acquired on a STELLARIS 8 confocal microscope using a 63× oil immersion objective lens and as a z-stack (z-step intervals: 0.8 μm). Displayed are Maximum Intensity Projections for visualization purposes in this figure (Images were processed as 3D images). (A) Maximum Intensity Projection of the whole image that was recorded. Most nuclei are adjacent to a ciliary signal, indicating a high ciliation rate. Scale bar: 20 μm. The ciliary channel (magenta) was segmented using the Otsu threshold algorithm. (B) Maximum Projection of a small inset, allowing to assess the segmentation quality. The segmentation mask (cyan) covers the whole ciliary signal, without including background pixels (oversegmentation), showing discontinuations within the ciliary masks (undersegmentation), or missing out cilia. Scale bar: 10 μm.

**Figure 7:**
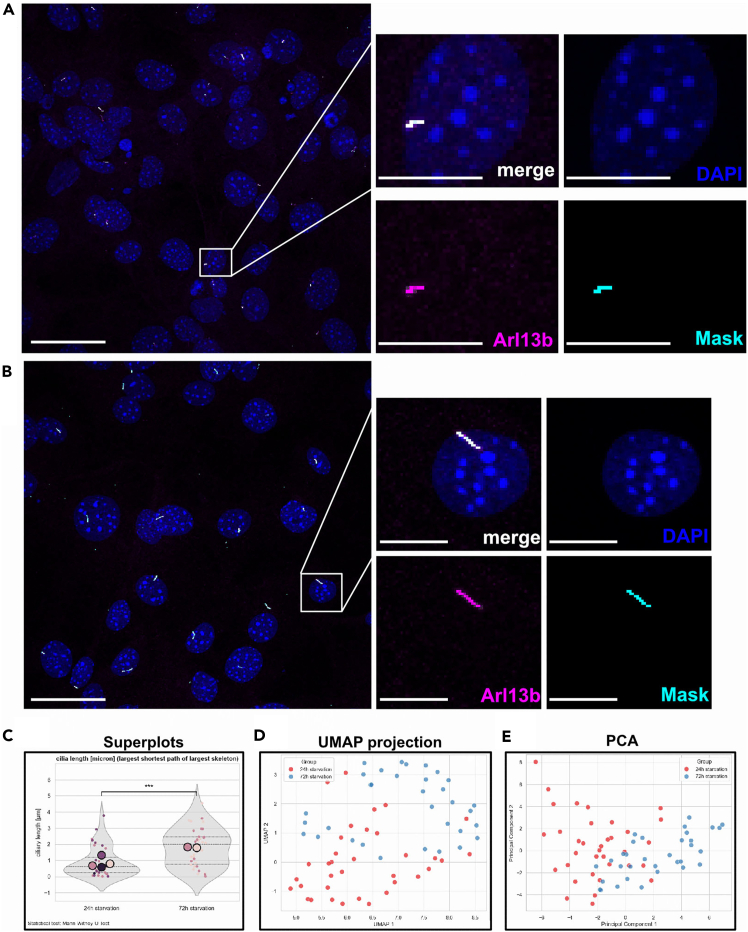
Prolonged serum starvation of 3T3 cells increases ciliary length and ciliation rate The ciliary signal was obtained from Arl13b-immunostaining. (A) Left: 3D image of 3T3-cells starved for 24 h. Most of the cells display short cilia. Scale bar: 40 μm. Segmentation of cilia was achieved using the Yen algorithm. Right: Magnified view allowing better assessment of segmentation quality; Ciliary masks completely cover Arl13b signal, without over- or undersegmentation. Scale bar: 10 μm. (B) Left: 3D image of 3T3-cells which were starved for 72 h. Almost all cells display cilia. Scale bar: 40 μm. Segmentation was achieved using the Yen algorithm. Right: Maximum Intensity Projection of a small inset to assess the segmentation quality in this image, indicative that the segmentation did not produce over- or undersegmented masks. (C) Ciliary length, plotted and statistically analyzed by CiliaQ and the CiliaQ Explorer. The superplot displays significant changes in ciliary length after 24 vs 72 h of starvation, determined by employing a Mann-Witney-U test (*p* < 0.001). Indicators for the replicate mean in the superplot scatter little, confirming high reproducibility of experiments and general applicability of selected settings. (D) Uniform Manifold Approximation and Projection (UMAP) embedding reveals that cilia from cells starved for 24 h (red) versus 72 h (blue) differ in their overall characteristics. This indicates that prolonged starvation leads to widespread changes in ciliary properties. Of note, UMAP embedding is not able to identify highly altered parameters, but provides an overview over the total pattern of all CiliaQ-derived measurements. The figure shows the first two dimensions of UMAP embedding. (E) Principal Component Analysis (PCA) segregates cilia after 24 h of starvation (red) from cilia starved for 72 h (blue). The plot visualizes Principal Component 1 and 2. For visualization purposes, A and B are displayed as Maximum Intensity Projections (Images were processed as 3D images).

**Figure 8:**
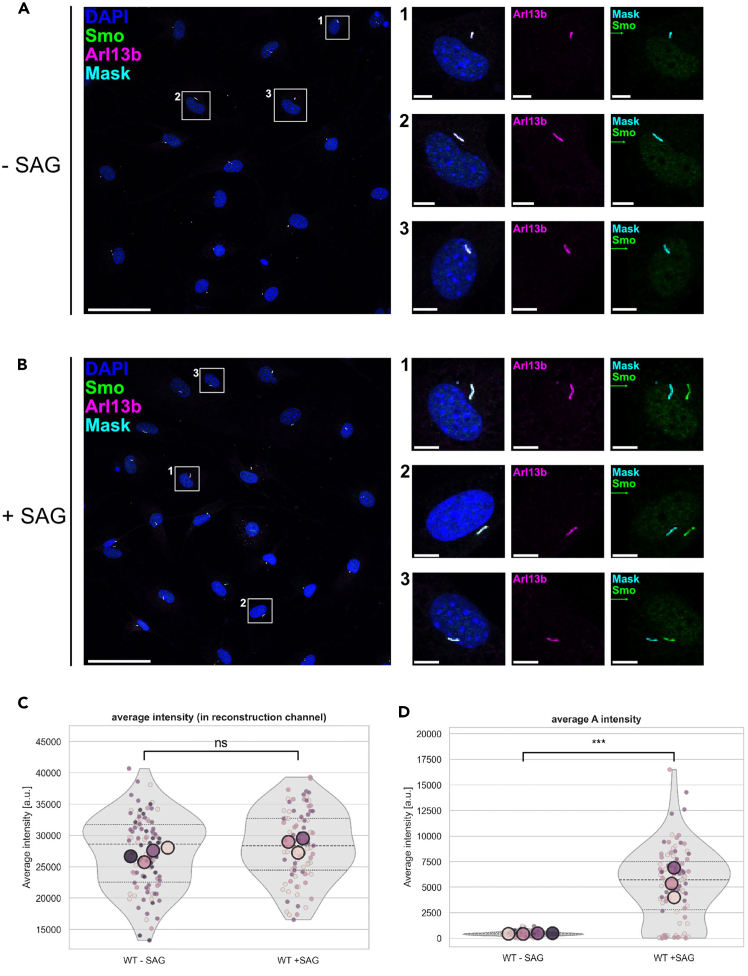
Smoothened (Smo) accumulates in primary cilia upon Smoothened agonist (SAG) treatment in primary adipoycte precursor cells Primary cilia were identified by Arl13b immunostaining and images were analyzed with CiliaQ (segmentation method: RenyiEntropy algorithm). Fluorescence intensities in the reconstruction channel (Arl13b) and Smo channel was quantified by CiliaQ within the boundaries of the ciliary masks. (A and B) Representative overview image of vehicle-treated (A) and 1μM SAG-treated (B) primary adipoycte precursor cells. Scale bar: 50 μm. Insets 1-3 show higher magnification views of individual cilia: overlap of Arl13b, DAPI, ciliary mask, and Smo (left), the Arl13b channel alone (middle), and overlay of the ciliary mask with Smo fluorescence intensity (right). In the right panel, the Smo channel was shifted 15 pixels to the right, to visualize colocalization of Smoothened and the ciliary mask. Scale bar: 5 μm. (C and D) Average Arl13b (C) and Smo (D) intensity as quantified by CiliaQ. CiliaQ Explorer reports that Smo intensity is significantly increased upon SAG-treatment (*p* < 0.001), while the average intensity in Arl13b intensity is not significantly changed (*p* = 0.14). Statistics determined by Mann-Witney-U test (C) and Student’s T-test (D), selected by CiliaQ Explorer based on the data distributions. The plot was generated using CiliaQ Explorer (v0.1.0).

When following this protocol, the user obtains a high-resolution, confocal image of a monolayer of mIMCD-3 ciliated cells, featuring a ciliation rate from up to 60%–70%. In this protocol, the imaging was performed using a Leica STELLARIS 8 point-scanning confocal microscope (Leica Microsystems, Wetzlar, Germany). However, this protocol can also be applied using other confocal microscopes that feature the imaging capabilities described in step 3–4. We provide examples for a good segmentation of mIMCD-3 cells (Figure 6), quantifying ciliary length changes in 3T3-cells (Figure 7) and quantifying ciliary Smoothened (Smo) signal in primary adipocyte precursor cells treated with Smo agonist (SAG)^5^ (Figure 8). Further image preprocessing, employing the “Subtract Background” or “Gaussian Blur” operation in the CiliaQ Preparator enables smoother segmentation masks and in turn, more accurate results (Figure 2D). Moreover, CiliaQ - if applied correctly using suitable image preprocessing and an appropriate segmentation algorithm - produces highly reproducible and reliable quantifications from images with segmented cilia, precisely measuring cilia length through skeletonizing the ciliary segmentation mask (Figure 2D). The skeletonization process can be supported through using the Gaussian blur function in CiliaQ itself which blurs the mask before skeletonizing it reducing side-branches in skeleton generation, which typically result from noisy cilia masks (Figure 2D). Apart from ciliary length, CiliaQ can measure a broad spectrum of different parameters describing cilium morphology and marker intensities (a description of all ciliary parameters can be found in the CiliaQ wiki). For all measured values, the CiliaQ Explorer software can generate superplots, a type of advanced categorial scatterplot, which is able to visualize the replicate identity of each data plot^6^ and thus allows to assess experimental reproducibility. Moreover, the CiliaQ Explorer allows visual assessment of the data distribution by overlaying a violin plot, also displaying both the median, as well as upper and lower quantiles (Figures 7C, 8C, and 8D). All plots are displayed both in the Jupyter Notebook and saved in an “Analysis” folder in the first directory that was inserted into the Explorer. More specifically, the generated “Analysis” folder contains two sub folders; the “Data” folder contains all summary .csv files and the “Plot” folder holds all plots generated during analysis.

Additionally, for quality control and identifying how different experimental groups of cilia discriminate, the CiliaQ Explorer displays similarity among all cilia in the data set through linear (Principal Component Analysis, PCA) and non-linear (Uniform Manifold Approximation and Projection, UMAP) dimensionality reduction methods applied on the CiliaQ measurement outputs (Figures 7D and 7E). Dimensionality reduction includes all CiliaQ-derived measurements despite ciliary ID, spatial features (x-, y-, and z-coordinate) and intensity thresholds for channel A, B, and for the basal stain, since these parameters do not encode ciliary properties but instead segmentation properties. Additionally, the dimensionality reduction algorithm neglects redundant measurements across the data. For instance, CiliaQ includes ciliary surface and volume in both microns and voxel units. Voxel-based units are neglected from analysis to avoid artificially inflating their influence on the dimension reduction. Dimensionality-reduction of the remaining output parameters enables to reveal patterns and relationship among the cilia from different conditions. In addition to comparing experimental groups, PCA and UMAP dimensionality reduction allows to reveal possible batch effects caused by individual replicates or to reveal technical artefacts or errors in individual images. Specifically, this is achieved by visually encoding individual replicates in a plot, which enables the user to identify outliers and verify consistency within each experimental group; e.g., replicates within experimental groups should cluster together while batch-to-batch or image-to-image variations should be less than differences in experimental groups.

In addition, CiliaQ Explorer employs Linear Discriminant Analysis (LDA),^7^ a supervised method for identifying CiliaQ measurements that contribute to class separation between experimental groups. This allows identification of highly variable features across groups (i.e., CiliaQ measurement parameters (e.g., cilia length) that discriminate groups well) in an automated way, eliminating the need to manually investigate each parameter to identify how cilia in different experimental groups distinguish from each other.

Moreover, the CiliaQ Explorer software performs quality control on each experimental group across the data (Figure 4A). More specifically, CiliaQ Explorer screens for outliers among all cilia regarding ciliary length, volume, maximum span, average cilia marker intensity and standard deviation, as well as the number of branches (note that normal cilia should optimally not display branches). Furthermore, the Explorer verifies that all experimental replicates are analyzed using the identical CiliaQ version. A summary of the quality control results is given by an appearing dialog and in the summary file, which also includes the identified cilia IDs. Thereby the program facilitates screening for segmentation imperfections and error backtracking to the ciliary images.

Note that quality control alerts do not necessarily prove that there are quality issues – they are meant to alert users to scrutinize whether segmentation is good and settings were appropriately selected. Also, a good quality control report is indicative for but not a proof for appropriate use of the software and good quality of segmentation as not all potential issues are detectable from CiliaQ output parameters or can be anticipated by the developers of CiliaQ Explorer.

Moreover, the procedure can be applied to microscopy images from varying microscope, ciliated cell types, magnification (including 40× magnification or water immersion objective lenses), ciliary marker staining (including acetylated αTubulin and genetically encoded cilia markers, enabling live cell imaging), or z-step intervals (0.2 μm/slice to 0.8 μm/slice). Please note that labeling using acetylated αTubulin might produce fluorescent signal at the ciliary base, requiring careful segmentation that incorporates the complete cilium into ciliary masks. Additionally, acetylated αTubulin is predominant in mitotic spindles resulting in potentially false positive mitotic spindles that need to be removed. With CiliaQ-deleteROI, a tool has been developed that manually assists in removing mitotic spindles from CiliaQ output files.^8^

## Quantification and statistical analysis

**Figure 9:**
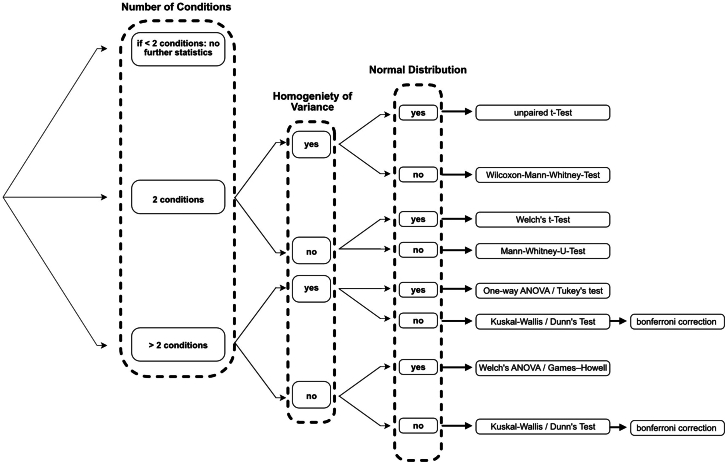
Overview of the decision algorithm implemented in the CiliaQ Explorer for statistical analysis The first decision algorithm determines the number of conditions, that are compared with each other. If only one condition is included into the analysis, there is no statistical analysis performed, else the algorithm continues with the second and third decision layer. In these layers, the CiliaQ Explorer software determines if the data features homogeneity of variance and if all conditions are normally distributed. Based on those results, the algorithm determines if the data features statistical significance by employing a pairwise (post-hoc) test.

The CiliaQ Explorer performs statistical analysis of the ciliary measurements, if selected during the setup process (step 14). For each CiliaQ output parameter, the algorithm by default checks if the data in each condition follows a normal distribution by employing Shapiro’s testing^9^ and if it features homogeneity of variance by performing Levene’s test.^6^ Based on those results and the number of analyzed conditions, the CiliaQ Explorer algorithm chooses the statistical test (and according post-hoc test if applicable) that is performed on the data. An overview of the decision algorithm can be found in Figure 9. summary of t-statistic, *p*-value, applied test, and significance level can also be found in the *statistics.txt* output document with ∗∗∗ for *p* ≤ 0.001, ∗∗ for *p* ≤ 0.01 and ∗ for *p* ≤ 0.05. The statistical analysis employed by the algorithm requires at least 3 cilia per condition. The statistical results are displayed in generated superplots as well. If the Explorer algorithm should not perform statistical analysis, the “Method for hypothesis testing” option can be set to “No statistics” during the Explorer setup process. In this case, the ciliary data could be exported to different data analysis software like MATLAB or GraphPad Prism by accessing it in the *summary.txt* output file.

## Limitations

The outcome of this protocol has limitations. First, the quality of the analysis highly depends on the parameters that have been chosen for imaging and analysis. This requires thorough inspection of the obtained segmentation after finishing step 12. Skipping this step might introduce a batch-dependent bias into the results. Since the CiliaQ algorithm for measuring ciliary parameters was refined in the last version updates, comparing data from different CiliaQ versions might introduce a batch dependent bias and affect the results. Hence, it is crucial that all analyses are performed on images analyzed by the same CiliaQ version and with the same imaging parameters.

In addition to CiliaQ, alternative tools to investigate cilia morphology have been developed.^10^^,^^11^^,^^12^ This protocol focuses on CiliaQ, because it enables highly accurate ciliary measurements by accurately skeletonizing the ciliary mask and thus accounting for ciliary bending and curvature. Moreover, CiliaQ performs a broad range of additional measurements, including ciliary volume, bending, average marker intensity and more. As a Fiji/ImageJ plugin, CiliaQ is freely available and open-source, can run on most computers (low and high cost, Linux, Mac, or Windows), is installable without any administrator rights, and provides a user-friendly and intuitive environment, making it broadly available and applicable.

Our Python-based pipeline can only be applied to CiliaQ outputs. Nevertheless, the underlying concept for data analysis and visualization, such as UMAP, PCA, and superplots, could be adapted to accommodate the output format of other tools.

The statistical analysis performed by the CiliaQ Explorer software has limitations: Including a large quantity of cilia (sample size *n*) into the same analysis may cause the statistical analysis to be significant even if the difference of the means is sparse. This might increase the risk of type I errors. Furthermore, the CiliaQ Explorer algorithm relies its choice of statistical tests solely on the number of experimental conditions, testing homogeneity of variance and testing normal distribution across the data. Although this could be sufficient for superficial statistical analysis,^13^^,^^14^ inflated type I errors have been reported in this context.^15^

In future versions of the CiliaQ Explorer, these limitations can be addressed by fitting more sophisticated models, such as Bayesian Statistics. By using credible intervals and probability distributions to determine the statistical validity of differences across experimental conditions, Bayesian Statistics potentially could reduce the risk of committing type I errors.^16^

Moreover, the decision tree to select the adequate statistical test is designed for continuous variables like ciliary length. However, ordinal CiliaQ-output variables such as the Shape Complexity Index, which is constrained to whole-integer values, would require the use of adapted statistical tests. Therefore, statistical test results of ordinal variables should be considered with care.

In summary, while CiliaQ Explorer suggests a statistical test and provides testing results for the suggested test, users are still required to assess whether the selected statistical test is applicable for their data and need to independently interpret their data.

## Troubleshooting

### Problem 1

The parameter of ciliary branches exceeds 1 for many cilia after CiliaQ analysis (related to step 14c.iii).

### Potential solution

Check ciliary masks for low signal to noise-ratio. In noisy cilia signal may result in an uneven outline of the cilia objects, inducing ciliary skeletons with multiple branches and thereby making it difficult to correctly trace the ciliary centerline from the skeleton. To avoid noisy ciliary masks, preprocess the ciliary signal using a Gaussian Blur. Insert the Gaussian Blur radius in the CiliaQ Preparator dialog (Figures 2D and 2E).

### Problem 2

The image contains large and strong areas of noise, producing false-positive signal during segmentation (related to step 10e).

### Potential solution

Consider using the “Subtract Background” function built into Fiji via “Process” > “Subtract Background” and apply it during CiliaQ Analysis (Figures 2D and 2E).

### Problem 3

Many cilia are excluded from the analysis (related to step 13a.v).

### Potential solution

Excluded cilia might touch the x-, y-, or z-border of the image. If many cilia touch the z-border, try recording a larger z-stack that covers a larger range in the z-dimensional space. There should be no ciliary signal on the first and last slice of the stack.

### Problem 4

Some cilia have no length value (related to step 13).

### Potential solution

Cilia with a 0 length measurement are often spherical and may represent ciliary stubs. Verify that segmentation is done properly and on the right channel. For example, if the ciliary analysis is accidentally performed on a channel containing basal body-signal, no length measurements could be expected.

### Problem 5

No threshold algorithm can generate high-quality cilium masks in highly heterogenous images (related to step 10g).

### Potential solution

If the ciliary signals vary across the image, try applying a “Subtract Background” method or splitting the image into smaller tiles and process every tile separately using an individual threshold algorithm. Thereby, the threshold is applied more regional and may be better adjusted to individual regions.

### Problem 6

ModuleNotFoundError when running the CiliaQ Explorer Pipeline in VS Code (related to step 14c).

### Potential solution

Ensure successful loading of the python dependencies in step 14a.viii. Also make sure that the Kernel suits the virtual environment.

### Problem 7

CiliaQ does not finish and runs “forever” (related to step 13b).

### Potential solution

Ensure that you selected the correct segmented channel for reconstruction in CiliaQ and that your segmentation is good (not containing too many background objects = not oversegmented).

## Resource availability

### Lead contact

Further information and requests for resources should be directed to and will be fulfilled by the lead contact, Nathalie Jurisch-Yaksi (nathalie.jurisch-yaksi@ntnu.no).

### Technical contact

Technical questions on executing this protocol should be directed to and will be answered by the technical contact, Daniel Burgdorf (daniel.burgdorf@uni-bonn.de).

### Materials availability

In this protocol, we did not generate new or exclusive materials. All used materials can be purchased and the software can be freely downloaded from the distributor.

### Data and code availability

As an open-source-Plugin for Fiji ImageJ, CiliaQ can be directly installed into ImageJ as described above. The python-based CiliaQ Explorer code is open-source, freely available at GitHub: https://github.com/burgdorfd/CiliaQ-Explorer and Zenodo: https://doi.org/10.5281/zenodo.15806534. The accession number for the images and CiliaQ output files reported in this paper is Zenodo: https://doi.org/10.5281/zenodo.16891093.

## Acknowledgments

We thank the Microscopy Core Facility of the Medical Faculty at the University of Bonn for providing support and instrumentation funded by the Bundesministerium für Bildung und Forschung (BMBF, Federal Ministry of Education and Research) – ACCENT: Förderung von Advanced Clinician Scientist im Bereich Immunopathogenese und Organdysfunktion and Gehirn und Neurodegeneration – Förderkennzeichen: 01EO2107 and the Deutsche Forschungsgemeinschaft (DFG, German Research Foundation) – project number 388159768. In addition, we thank David U. Mick for kindly providing the 3T3-L1 cilia-iAPEX cells used in this work.

This work was funded by the 10.13039/501100001659Deutsche Forschungsgemeinschaft (DFG, Germany Research Foundation), FOR5547—project ID 503306912 (to N.J.-Y. and D.W.), DFG-WA3382/8-1 (to D.W.), SFB 1454—project ID 432325352 (to D.W.), Germany’s Excellence Strategy—EXC2151—project ID 390873048 (to D.W.), a grant from the 10.13039/501100003042Else Kröner Fresenius Foundation (2021.EKFSE.53), and The 10.13039/501100005416Research Council of Norway (FRIPRO grant 314189, N.J.-Y.). J.N.H. was supported by an 10.13039/100004410EMBO Postdoctoral Fellowship (ALTF 556-2022). The graphical abstract and Figure 1 were created with BioRender.com.

## Author contributions

D.B.: coding and writing; S.Y.: writing and image acquisition; K.S.: image acquisition; D.W. and N.J.-Y.: supervision and funding acquisition; J.N.H. and N.J.-Y.: code design, reviewing, and editing; all authors: reviewing and editing manuscript text.

## Declaration of interests

The authors declare no competing interests.

## Supplemental information

Table S1. Spreadsheet for tracking experimental metadata, containing fields for important experimental information to ensure reproducibility across multiple experimental runs, related to step 8
